# Draft Genome Sequence of *Tenacibaculum soleae* UCD-KL19

**DOI:** 10.1128/genomeA.01120-16

**Published:** 2016-10-13

**Authors:** Karley M. Lujan, Jonathan A. Eisen, David A. Coil

**Affiliations:** a Davis Genome Center, University of California Davis, Davis, California, USA; b Department of Evolution and Ecology and Department of Medical Microbiology and Immunology, University of California Davis, Davis, California, USA

## Abstract

Here, we present the draft genome sequence of *Tenacibaculum soleae* strain UCD-KL19. The assembly contains 3,012,701 bp in 46 contigs. This strain was isolated from a seagrass leaf (*Zostera marina*) collected from Bodega Bay, CA, as a part of an undergraduate student research project on isolating bacteria from seagrass.

## GENOME ANNOUNCEMENT

Some species within the genus *Tenacibaculum* are known fish pathogens that cause tenacibaculosis, an infectious disease that impacts farmed marine finfish all across the world (1). *Tenacibaculum soleae* is a Gram-negative rod-shaped bacterium with gliding motility, belonging to the family *Flavobacteriaceae* (2).

*T. soleae* UCD-KL19 was isolated from a phosphate-buffered saline (PBS) rinse of a seagrass leaf as a part of the Seagrass Microbiome Project (https://seagrassmicrobiome.org/), with a focus on culturing bacterial isolates. The PBS rinse of the seagrass leaf was cultured on a Difco marine broth agar plate at 25°C for two days. A single colony was selected for two rounds of dilution streaking, and one of the resulting colonies was used to make an overnight culture in liquid Difco marine broth at 25°C. Genomic DNA was extracted using a Promega Wizard genomic DNA purification kit. After the extraction, PCR amplification of the 16S rRNA gene was undertaken using the 27F and 1391R primers. This PCR product was Sanger sequenced, and the resulting consensus sequence was analyzed using BLAST (3). Using the Ribosomal Database Project (RDP) (4), an alignment of this isolate and other *Tenacibaculum* isolates was created (4). This alignment was used to infer a maximum-likelihood phylogenetic tree, which was created in FastTree (5) and viewed in Dendroscope (5, 6). This isolate was found within a well-supported monophyletic clade that contained several other *T. soleae* strains that did not occur elsewhere in the tree. In order to complete the whole-genome sequencing, a paired-end library was created using a Nextera XT library preparation kit (Illumina). Using a PippinPrep (Sage Science), we selected 600- to 900-bp fragments. The size-selected library was sequenced on a paired-end 300-bp run of an Illumina MiSeq. Following the completion of quality trimming and error correction by the A5-miseq assembly pipeline (7, 8), 1,066,673 quality reads assembled into 26 scaffolds, with an estimated 87× coverage and a G+C content of 30.3% (8, 9). PhyloSift (7) was used to estimate genome completeness by searching for a list of 37 highly conserved single-copy marker genes, and a single copy of each one was present in this assembly.

Annotation was completed using RAST (10). The annotated *T. soleae* strain UCD-KL19 genome contains 2,812 coding sequences and 54 noncoding RNAs. The full-length 16S sequence (1,515 bp) from RAST was used in a BLAST search, which revealed 100% identity to a *T. soleae* strain. This same 16S sequence was then used to build a second phylogenetic tree using the methods described above (https://dx.doi.org/10.6084/m9.figshare.3469766.v1). As described above, *T. soleae* strain UCD-KL19 was located in a monophyletic clade of *T. soleae* strains.

### Accession number(s).

This whole-genome shotgun project has been deposited at DDBJ/ENA/GenBank under the accession no. MAKX00000000. The version described in this paper is version MAKX01000000.
